# Botulism outbreak in a rural Ethiopia: a case series

**DOI:** 10.1186/s12879-021-06969-w

**Published:** 2021-12-20

**Authors:** Tigist Bacha, Ermias Abebaw, Ayalew Moges, Amsalu Bekele, Afework Tamiru, Ishmael Shemsedin, Dawd S. Siraj, Daddi Jima, Wondwossen Amogne

**Affiliations:** 1 Department of Pediatrics and Child Health, Saint Paul, Millennium Medical College, Addis Ababa, Ethiopia; 2 Department of Internal Medicine, College of Health Sciences, School of Medicine, Addis Ababa University, Addis Ababa, Ethiopia; 3 Department of Public Health, Institute of Health Sciences, Wollega University, Nekemte, Ethiopia; 4 Department of Emergency Medicine and Critical Care, Saint Paul, Millennium Medical College, Addis Ababa, Ethiopia; 5 Division of Infectious Diseases, University of Wisconsin-Madison School of Medicine and Public Health, Madison, USA; 6 Ethiopian Public Health Institute, Addis Ababa, Ethiopia; 7 Department of Pediatrics and Child Health, College of Health Sciences, Addis Ababa University, Addis Ababa, Ethiopia

**Keywords:** *Clostridium botulinum*, Foodborne diseases, Outbreak, Ethiopia

## Abstract

**Background:**

Foodborne botulism, a toxin-mediated illness caused by *Clostridium botulinum,* is a public health emergency. Types A, B, and E *C. botulinum* toxins commonly cause human disease. Outbreaks are often associated with homemade and fermented foods. Botulism is rarely reported in Africa and has never been reported in Ethiopia.

**Case presentation:**

In March 2015, a cluster of family members from the Wollega, Oromia region, western Ethiopia presented with a symptom constellation suggestive of probable botulism. Clinical examination, epidemiologic investigation, and subsequent laboratory work identified the cause of the outbreak to be accidental ingestion of botulinum toxin in a traditional chili condiment called “Kochi-kocha,” cheese, and clarified butter. Ten out of the fourteen family members who consumed the contaminated products had botulism (attack rate 71.4%) and five died (case fatality rate of 50%). Three of the patients were hospitalized, they presented with altered mental status (n = 2), profound neck and truncal weakness (n = 3), and intact extremity strength despite hyporeflexia (n = 3). The remnant food sample showed botulinum toxin type A with mouse bioassay and *C. botulinum* type A with culture. Blood drawn on day three of illness from 2/3 (66%) cases was positive for botulinum toxin type-A. Additionally, one of these two cases also had *C. botulinum* type A cultured from a stool specimen. Two of the cases received Botulism antitoxin (BAT).

**Conclusion:**

These are the first confirmed cases of botulism in Ethiopia. The disease occurred due to the consumption of commonly consumed homemade foods. Definite diagnoses of botulism cases are challenging, and detailed epidemiologic and laboratory investigations were critical to the identification of this case series. Improved awareness of botulism risk and improved food preparation and storage may prevent future illnesses. The mortality rate of botulism in resource-limited settings remains high. Countries should make a concerted effort to stockpile antitoxin as that is the easiest and quickest intervention after outbreak detection.

## Background

Foodborne botulism, a toxin-mediated illness caused by *C. botulinum,* is a public health emergency. *C. botulinum* is a heterogeneous group of gram-positive, rod-shaped, spore-forming, obligate anaerobic bacteria. It is ubiquitous and easily isolated from the surfaces of vegetables, fruits, and seafood, and exists in soil and marine sediment worldwide. The bacterium produces lethal toxins types A, B, E, and F [1–3].

*Clostridium botulinum* toxins cause human disease, with toxin type A accounting for over half of all cases [4, 5]. Other *Clostridium* species such as toxigenic strains of *Clostridium baratii*, *Clostridium butyricum*, and *Clostridium argentinensis less commonly produce botulinum neurotoxins *[6]*.* There are multiple transmission mechanisms. Foodborne botulism happens when *C. botulinum* grows and produces toxins in food before consumption. Unlike foodborne botulism, infant botulism occurs when infants ingest *C. botulinum* spores, which germinate in the gut and release toxins. Wound botulism is rare and occurs when spores get into an open wound. Inhalation botulism is associated with accidental or intentional events (such as bioterrorism), resulting in the release of toxins in aerosols. There is no human-to-human transmission of botulism [3].

Botulism outbreaks are often associated with improperly processed homemade and fermented foods. Diagnosis is made in a cluster of cases with acute, bilateral cranial neuropathies and symmetrical descending motor weakness without fever and loss of consciousness. Mouse bioassay is the gold standard for diagnosis [7].

Foodborne botulism is rarely reported in Africa and never before in Ethiopia. It is due to its rare occurrence, low clinical index of suspicion, and limited diagnostic capacity [1, 8].

Kenya reported two foodborne botulism outbreaks that occurred 1 year apart. The first cases were reported among Kenyan nomads in 1978 when a *C. botulinum* TypeA outbreak from traditionally prepared milk caused six deaths [9]. One year later, another outbreak in Kenya was caused by eating contaminated termites [8], and five of six cases were fatal. In April 1991, over 90 patients were admitted to the Cairo hospital with symptoms consistent with botulism [10]. Samples of implicated food subsequently grew *C. botulinum*. Eighteen of the patients (20%) died of the illness. In February 2002, two siblings (aged eight and 12 years old) from South Africa developed acute flaccid paralysis and died. The implicated cause was a preformed toxin in canned food that was commercially produced in South Africa [11]. In October 2008, botulism was reported in three Ugandan boarding-school students [12]. Morocco reported 15 cases of food-borne botulism in 1999 [13]. Finally, a suspected outbreak of botulism affecting three patients in a family of six was reported in Nigeria after the family ate a home-cooked meal of pounded yam with fresh fish pepper soup. The clinical presentation of the three affected cases was consistent with botulinum poisoning. Two of the affected cases died. The third case survived after the administration of appropriate antitoxin. Samples were tested but did not show *C. botulinum* [1].

From 1986 to 2015, 466 confirmed cases of botulism were recorded in Italy (421 were food-borne). The most frequently seen form of botulism is due to the consumption of improperly canned foods at home, and 50% of such cases were registered in Europe. Itsuggests the need to raise public awareness of the risk of foodborne botulism, especially concerning home-preserved foods, as well as improving the training of health providers to ensure a quick and accurate diagnosis of botulism [14]. This case series describes an outbreak of foodborne botulism in a rural community from the west Wollega, Oromia region, western Ethiopia.

*A case of botulism* is defined as symptoms, signs, and neuro-diagnostic studies consistent with botulism [9]. Cases symptoms were identified through interactive interviews, medical record reviews, and systematic clinical examinations and by accounts of symptoms reported by family members in the cases of deaths before the investigation began. Cases with atypical presentations for botulism were evaluated with electrodiagnostic and cerebrospinal fluid (CSF) testing to determine if findings were consistent with an alternative illness. Laboratory diagnostic testing was performed on biological specimens and food samples.

*Confirmed cases* are defined as botulinum toxin is detected from a biologic specimen (serum or stool) or *C. botulinum* is cultured from stool. Serum, stool, and food samples were tested by mouse bioassay and mass spectrometry for different types of botulinum neurotoxin (BoNT); food and stool samples were cultured for isolation of botulinum toxin-producing clostridium. BoNTs are synthesized mostly by *Clostridium* species named *Clostridium botulinum* and more rarely by other *Clostridium* species such as toxigenic strains of *Clostridium baratii* type F, *Clostridium butyricum* type E, and *Clostridium argentinensis* [15, 16]. Botulinum isolates were further characterized by Whole genome sequencing (WGS).

These case definitions were set in line with the Ethiopian Public Health Emergency Management guidelines developed by the Ethiopian Public health institute (EPHI). EPHI is the institution responsible for dealing with outbreaks/surveillance in Ethiopia [17]. The Oromia Regional State Health Bureau and EPHI, together with US Center for Disease Prevention and Control (CDC), investigated the described outbreak. The cases were reported from Oromia Region, West Wollega, West Ethiopia.

## Case presentation

### Description of the outbreak

The outbreak occurred in a family of 14 members after a celebration where homemade cultural food was served. The first reported onset of symptoms (n = 2 cases) was on March 8, 2015, one day after the ceremony. A cluster of 10 cases of probably 14 family members fulfilled the clinical diagnostic criteria with an attack rate of 71.4% (Table 1). The median age of the cluster was 19 years old (range 4–45 years) and six (60%) were female. The onset of symptoms was marked with fatigue, headache, dysphagia, vomiting, blurred vision, and slurred speech. Physical findings included bradycardia, cranial nerve palsies (ptosis, dysarthria, dysphonia, dysphagia), and bilateral upper and lower extremity weakness. Five of the ten symptomatic cases died (case fatality rate 50%); three died before they arrived at district hospital (one at home and two on the way to the hospital), two died at the district hospital where intensive care unit (ICU) and mechanical ventilators were not available. Three cases were referred to a tertiary care facility in Addis Ababa where two were admitted to the ICU; one required mechanical ventilator support. The remaining six (2 symptomatic and 4 asymptomatic) were observed at the district hospital and discharged.

**Table 1:** Characteristics of patients with botulism and final outcomes, West Wollega, Ethiopia

Patient	Age (years)	Sex	IP* (days)	Type A toxin in the		Stool culture	EMG	Outcome
				Serum	Stool			
1	4	Male	3	Negative	Negative	Negative	Positive	Survive^c^
2	6	Male	2	Positive	Negative	Negative	Not done	Survive^b^
3	8	Female	3	Negative	Negative	Not done	Not done	Survive^b^
4	16	Female	2	Not done	Not done	Not done	Not done	Died^a^
5	18	Female	2	Not done	Not done	Not done	Not done	Died^b^
6	20	Female	3	Not done	Not done	Not done	Not done	Died^a^
7	21	Male	1	Not done	Not done	Not done	Positive	Survive^a^
8	25	Female	1	Not done	Not done	Not done	Not done	Died^a^
9	40	Female	2	Positive	Not done	Positive	Positive	Survive^c^^**^
10	45	Male	3	Not done	Not done	Not done	Not done	Died^b^

March 2015

NB: ^*^Incubation Period, ^a^Home, ^b^District hospital, ^c^Tertiary hospital, ^**^Required mechanical respiration

### Clinical manifestations

The clinical data are summarized in (Table 2). The onset was abrupt. Symptoms with the highest frequency were fatigue (9/10), difficulty opening eyes (9/10), headache (8/10), dysphagia (8/10), slurred speech (7/10), visual disturbance (7/10), difficulty supporting the head (7/10), and vomiting (6/10). Those who were hospitalized demonstrated altered mental status (n = 2), profound neck and truncal weakness (n = 3), and intact extremity strength (n = 3). Additionally, they had hyporeflexia and autonomic findings including diarrhea and reactive pupils without mydriasis (n = 1). A 4-year-old child who survived the illness had a classic presentation of descending paralysis that started with headache, vomiting, and blurred vision, followed by dysphagia and upper and lower extremity weakness. An adult case, which required mechanical ventilation also, had classic descending paralysis. Two adult ‘patients’ received 20 mL of BAT as an intravenous I.V. infusion.

**Table 2:** Distribution of symptoms and signs of Botulism cases, West Wollega, Ethiopia, March 2015

Neurologic symptoms and signs	Frequency	Percentage (%)
Headache	8	80
Dysphagia	8	80
Dysarthria	7	70
Ptosis	7	70
Diplopia	7	70
Profound neck and truncal weakness	7	70
Pupils constricted	4	40
Upper extremity weakness	3	30
Intact extremity strength	3	30
Diminished gag	3	30
Ophthalmoparesis	3	30
Facial paresis	3	30
Deep tender reflexs diminished or absent	3	30
Altered mental status	2	20
Nystagmus	2	20
Dyspnea	1	10
Gastrointestinal symptoms and signs		
Vomiting	6	60
Constipation	4	40
Increased salivation	2	20
Others		
Fatigue	9	90
Sore throat	8	80
Dizziness	4	40
Bradycardia	2	20

Among the survivors, three of the patients with severe disease were referred to tertiary care. Of those, one adult patient was discharged with residual weakness of the extremities. The 4 year old child had improved body weakness but had still bilateral ptosis. The third patient's muscle weakness fully recovered, returning to a fully functional status. Six patients, who were observed at a nearby district hospital for a few days were discharged with no neurologic sequelae or functional abnormalities.

### Clinical laboratory testing

Serum and stool samples were tested by mouse bioassay and mass spectrometry for botulinum neurotoxin. Food and stool samples were cultured for isolation of *C. botulinum*. Foods suspected to be contaminated by the toxin and accessible at the affected households were collected; minimal gelatin phosphate buffer was added and the specimens with detected botulinum toxin were cultured for botulinum toxin-producing clostridia. Blood drawn on the third day of illness from two of the cases was positive for botulinum toxin type A. One of the two cases also had *C. botulinum* type A cultured from a stool specimen.

Three patients had electromyography (EMG) that confirmed neuromuscular dysfunction severely affecting the face and proximal upper extremity muscles. The 4 year old child had a lumbar puncture with CSF that showed 3 nucleated cells, protein = 37.7 gm/dl, glucose = 98 mg/dl, Gram stain, and culture showed no organism.

### Food vehicle investigation

An extensive dietary history was taken in all cases with a clinical diagnosis of botulism. The extended family shared a common food source and were served from the same plate. One day before the first onset of symptoms, nine of the family members with clinical diagnosis ate sorghum porridge with cheese and yogurt for lunch. The cheese was kept for an indefinite time in a tightly sealed container. A traditional food called “kochi-kocha” and butter were added to the cheese prior to eating. One family member ate the kochi-kocha with bread (no sorghum porridge) and did not get ill. The chili condiment “Kochi-Kocha” (which is made from fresh green chili, clarified butter, ginger, garlic, salt, “Besobila^1^” and “Tenaadam^2^”), and clarified butter were both kept at room temperature in airtight sealed plastic containers separately for an average of 3 days. On the same day, all household members ate Injera (a national staple made of fermented Teff) and bean stew prepared with clarified butter for breakfast and dinner.

### Food vehicle laboratory testing

The testing revealed *C. botulinum* toxin from the chili condiment “Kochi-Kocha” and the clarified butter. The “Kochi-Kocha” was also positive by mouse bioassay for botulinum toxin type A. A very tiny remnant of clarified butter that was collected from the empty container was found to be weakly positive. *C. botulinum* type A was cultured from two dried specimens which contain milk and cheese collected from gourd containers (Table 3).

**Table 3:** Food laboratory testing result of the Botulism cases, West Wollega, Ethiopia March 2015

Food type	Result
Bean stew	Negative
Chili condiment, “Kochi-Kocha”	botulinum toxin type A
Edible oil	Negative
Clarified butter remnants in a blue sealable plastic container	Probable weak botulinum toxin type Anal
Dried milk from a one gourd	Culture positive for C. botulinum type A, unable to test for the presence of toxin due to the quantity of specimen
Dried cheese from second the gourd	Culture positive for C. botulinum type A, unable to test for the presence of toxin due to the quantity of specimen
Sorghum porridge	Negative

The clinical specimens and food specimens, i.e., dried cheese, dried milk, clarified butter, and a chili condiment, contained either BoNT type A or *C. botulinum* type A. The A2 subtype of the BoNT gene was identified in all *C. botulinum* isolates. Whole genome sequencing and single nucleotide polymorphism (SNP) analysis showed that the isolates from the samples (both clinical sample and food sample) are related and have a common ancestor. However, it should be noted that the result couldn’t definitively confirm the source of the spores as there was a possibility of cross-contamination during food preparation.

### Public health measures

All food items used in the preparation of the implicated meal served in the ceremony were removed from the house and destroyed. The remaining family members were oriented to appropriately clean utensils in the house before using them for food preparation. Heath extension workers in the area were trained about the natural reservoir, mode of transmission, the natural history of *C. botulinum,* and how it can be prevented so that they will promote awareness in their regular communication with the community.

## Discussion and conclusion

This case report describes the first proven cases of foodborne botulism in Ethiopia. The outbreak was caused by toxin type A botulinum toxin. The case fatality rate of the outbreak was 50% and the attack rate was 71.4%. This is similar to other case reports of foodborne botulism reported elsewhere [9, 12, 13, 18, 19]. Botulinum toxin is a highly poisonous neurotoxin synthesized by the bacteria *C. botulinum*. The initial consideration of botulism based on its clinical presentation and clustering of the cases is the most important step to diagnosis and quickly managing clinical care.

Symptoms of foodborne botulism usually begin 12 to 36 h after ingestion of the preformed toxin [7]. In our cases, the incubation period varied between 1 to 3 days. Incubation periods as long as 1 week are seen when the volume of the preformed toxin inoculum is small. Symptoms predominantly include the involvement of the gastrointestinal tract and the central nervous systems [20]. As recognized during the previous foodborne botulism outbreaks, the attack rate is not 100% [19, 21]. In our case series, the attack rate of 70% among those who ate is explained by an uneven distribution of the toxin in the food, a dose–response relationship, or unrecognized host factors that confer resistance to the toxin.

Clinical presentation and demonstration of toxin in the blood, stool, vomitus of a suspected case, or food items help to make the diagnosis of foodborne botulism [13]. In this outbreak, as is common in foodborne botulism, diarrhea was a common early symptom [2]. Diarrhea is caused by toxins other than botulinum toxin including bacteria and/or viruses contaminating the same source of food. Pupils could be reactive without mydriasis, as in the described cases, because the parasympathetic fibers in the oculomotor nerve were spared. Altered mental status in cases of botulism could be due to lack of adequate oxygenation, infection, or other metabolic disorders [22].

Clinical signs and symptoms and epidemiologic investigation of cases are critical, especially when laboratory test results may be delayed or unavailable. The differential diagnosis of food-borne botulism includes myasthenia gravis, tick paralysis, Guillain–Barre syndrome, poliomyelitis, stroke, and heavy metal intoxication [4].

In our report, all cases consumed cheese with “Kochi-Kocha,” which was made from chili and butter. Botulinum toxin type A was identified from the cheese, butter, and chili. All foods were stored in sealed containers which can create an oxygen-free environment, thereby favoring the germination of clostridium. Fats can provide the low acidity environment necessary for anaerobic bacteria need for spore germination and toxin elaboration. The plastic sealed containers facilitate the anaerobic environment. The chili is grown in soil and can harbor bacterial spores. There was also the possibility of cross-contamination of toxins while serving the food.

Due to the limited diagnostic capacity, the prevalence of the disease in Ethiopia is not well known. Early recognition of botulism is critical for the treatment of cases and to limit the extent of the outbreak by potentially identifying the possible contaminated food sources. The mainstay of treatment is respiratory support with over 34% requiring mechanical ventilation [5, 14], a major limitation in Ethiopia. Botulism antitoxin (BAT) when given early in the disease process can neutralize circulating botulinum toxin, stopping the further progression of the disease. Unfortunately, antitoxin does not reverse the damage that has already been occurred [23]. Unfortunately, BAT is not readily available in Ethiopia.

Our investigation has limitations. Investigators were not able to interview or examine all the cases. It was also not possible to collect biological samples from all of the cases. The informants about the symptoms of the cases might have recall bias.

The mortality rate of botulism in developing countries with limited resources remains high. Important contributing factors include delay in diagnosis, lack of antitoxin, and the limited presence of an intensive care support system that providers for ventilator support. Countries should make a concerted effort to stockpile antitoxin, as that is the easiest and quick way of intervening upon outbreak detection.

This outbreak investigation can be considered a baseline for public health teams on how to detect and respond to similar outbreaks in the future. At the Ministry of Health and Regional Health Bureau level, a combined strategy of disease surveillance, recognition, confirmation, and treatment has to be devised to assess the extent of botulism and develop prevention strategies for this newly confirmed disease in Ethiopia. There should be a strong partnership between clinicians and public health authorities at all levels for rapid detection of possible clusters of botulism and complete investigation to identify the primary source of infection and the specific process of the risk introduction of the bacilli into the implicated food. Efforts should also be made to inform communities about the risk of food storage practices and the types of food liable for spore contamination. Systematic ongoing surveillance using standardized botulism case definition must be introduced and integrated with the existing acute flaccid paralysis surveillance system. It is also essential to establish a system of referral and logistics where patients suspected of botulism could easily be transported for inpatient admission to provide care such as mechanical ventilation, botulism antitoxin, and nutritional rehabilitation.

## Data Availability

The data sets used and/or analyzed during the current study are available from the corresponding author on reasonable request. Data is archived from the Ethiopian Public health institute.

## Abbreviations

BAT: Botulism antitoxin
CDC: Center for disease control and prevention
CSF: Cerebrospinal fluid
EMG: Electromyography
EPHI: Ethiopian Public health Institute
ICU: Intensive Care Unit
WGS: Whole genome sequencing

## Footnotes

1 A local herb whose scientific name is *Ocimum sanctum/tenuiflorum*.

2 Another local herb is considered to have medicinal value and is used as a flavoring additive. Its scientific name is *Rutachalepensis L.*

## References

[1] Okunromade O, Dalhat MM, Umar AM, Dada AO, Nikau J, Maneh L, Ita OI, Balogun MS, Nguku P, Ojo O, Ihekweazu C (2020). Emergency response to a cluster of suspected food-borne botulism in Abuja, Nigeria: challenges with diagnosis and treatment in a resource-poor setting. Pan Afr Med J.

[2] Dowell VR (1984). Botulism and tetanus: selected epidemiologic and microbiologic aspects. Rev Infect Dis.

[3] https://www.who.int/news-room/fact-sheets/detail/botulism. Accessed 7 Apr 2021.

[4] Sobel J (2005). Botulism. Clin Infect Dis.

[5] Fleck-Derderian S, Shankar M, Rao AK, Chatham-Stephens K, Adjei S, Sobel J, Meltzer MI, Meaney-Delman D, Pillai SK (2017). The epidemiology of foodborne botulism outbreaks: a systematic review. Clin Infect Dis..

[6] Rasetti-Escargueil C, Lemichez E, Popoff MR (2020). Toxemia in human naturally acquired botulism. Toxins.

[7] Bleck TP, Hodowanec A. Botulism (*Clostridium botulinum*). Principles and Practice of Infectious Diseases, 8th ed. In: Mandel GL, Bennett JE, Dolin R, editors. Churchill Livingstone, Philadelphia; 2016. pp. 2762

[8] Nightingale KW (1980). Ayim EN Outbreak of botulism in Kenya after ingestion of white ants. Br Med J.

[9] Smith DH, Timms GL, Refai M (1979). Outbreak of botulism in Kenyan nomads. Ann Trop Med Parasitol.

[10] Weber JT, Hibbs RG, Darwish A, Mishu B (1993). A massive outbreak of type E botulism associated with traditional salted fish in Cairo. Infect Dis.

[11] Frean J, Arntzen L, Van den Heever J, Perovic O (2004). Fatal type A botulism in South Africa, 2002. Trans R Soc Trop Med Hyg.

[12] Viray MA, Wamala J, Fagan R, Luquez C (2014). Outbreak of type A botulism in a boarding school-Uganda, 2008. Epidemiol Infect.

[13] Kissani N, S Moutawakkil, A Chakib, I Slassi Fifteen cases of food-borne botulism in Morocco: significant diagnostic contribution of electrodiagnosis Rev Neurol (Paris). 2009;165(12):1080-5. 10.1016/j.neurol.2009.03.014 10.1016/j.neurol.2009.03.014 19709701

[14] Anniballi F, Auricchio B, Fiore A, Lonati D, Locatelli CA, Lista F, Fillo S, Mantaldarino G, De Medici D (2017). Botulism in Italy, 1986 to 2015. Euro Surveill.

[15] Public Health Agency of Canada. Botulism. In: Case Definitions for Communicable Diseases under National Surveillance. Canada Communicable Disease Report. 2009; 35S2.

[16] Meng X, Karasawa T, Zou K, Kuang X, Wang X, Lu C, Wang C, Yamakawa K, Nakamura S (1997). Characterization of a neurotoxigenic *Clostridium butyri*cum strain isolated from the food implicated in an outbreak of food-borne type E botulism. J Clin Microbiol.

[17] Public Health Emergency Management Guidelines for Ethiopia; 2012. pp. 48–65. https://ephi.gov.et/images/guidelines/phem-guideline-final.pdf. Accessed 12 Aug 2021.

[18] Angulo FJ, Getz J, Taylor JP (1998). A large outbreak of botulism: the hazardous baked potato. J Infect Dis.

[19] Townes JM, Cieslak PR, Hatheway CL (1996). An outbreak of type A botulism associated with commercial cheese sauce. Ann Intern Med.

[20] Smith GR, Turner A (1987). Factors affecting the toxicity of rotting carcasses containing *Clostridium botulinum* type C. Epidemiol and Infect.

[21] Varma JK, Katsitadze G, Moiscrafishvili M (2004). Signs and symptoms predictive of death in patients with foodborne botulism- Republic of Georgia, 1980–2002. Clin Infect Dis.

[22] August M, Hamele M (2020). Two cases of infant botulism presenting with altered mental status Hawaii. J Health Soc Welf.

[23] Kalluri P, Crowe C, Reller M, Gaul L (2003). An outbreak of foodborne botulism associated with food sold at a salvage store in Texas. Clin Infect Dis.

